# Identification of Compounds That Inhibit Estrogen-Related Receptor Alpha Signaling Using High-Throughput Screening Assays

**DOI:** 10.3390/molecules24050841

**Published:** 2019-02-27

**Authors:** Caitlin Lynch, Jinghua Zhao, Srilatha Sakamuru, Li Zhang, Ruili Huang, Kristine L. Witt, B. Alex Merrick, Christina T. Teng, Menghang Xia

**Affiliations:** 1 National Center for Advancing Translational Sciences, National Institutes of Health (NIH), Bethesda, MD 20814, USA; caitlin.lynch@nih.gov (C.L.); jinghua.zhao@nih.gov (J.Z.); sakamurus@mail.nih.gov (S.S.); li.zhang6@nih.gov (L.Z.); huangru@mail.nih.gov (R.H.); 2 Division of the National Toxicology Program, National Institute of Environmental Health Sciences, NIH, Research Triangle Park, NC 27709, USA; witt@niehs.nih.gov (K.L.W.); merrick@niehs.nih.gov (B.A.M.)

**Keywords:** ERRα, endocrine disruption, bioenergetic signaling pathways, mechanism of action, cancer

## Abstract

The nuclear receptor, estrogen-related receptor alpha (ERRα; NR3B1), plays a pivotal role in energy homeostasis. Its expression fluctuates with the demands of energy production in various tissues. When paired with the peroxisome proliferator-activated receptor γ coactivator 1α (PGC-1α), the PGC/ERR pathway regulates a host of genes that participate in metabolic signaling networks and in mitochondrial oxidative respiration. Unregulated overexpression of ERRα is found in many cancer cells, implicating a role in cancer progression and other metabolism-related diseases. Using high throughput screening assays, we screened the Tox21 10K compound library in stably transfected HEK293 cells containing either the ERRα-reporter or the reporter plus PGC-1α expression plasmid. We identified two groups of antagonists that were potent inhibitors of ERRα activity and/or the PGC/ERR pathway: nine antineoplastic agents and thirteen pesticides. Results were confirmed using gene expression studies. These findings suggest a novel mechanism of action on bioenergetics for five of the nine antineoplastic drugs. Nine of the thirteen pesticides, which have not been investigated previously for ERRα disrupting activity, were classified as such. In conclusion, we demonstrated that high-throughput screening assays can be used to reveal new biological properties of therapeutic and environmental chemicals, broadening our understanding of their modes of action.

## 1. Introduction

The estrogen-related receptor alpha (ERRα) was discovered based on the structural similarity to the estrogen receptor alpha (ERα); however, it is important to note that ERRα does not bind estrogen [1]. The potential for cross-talk between these two receptors was evident by the discovery that they share a similar hormone response element, thereby interfering with each other’s activities [2,3,4,5]. After in-depth analyses of the ERα and ERRα binding site specificities and their target genes, it was discovered that the prime functions of these two receptors were distinct [6,7,8]. The expression pattern of ERRα in various tissues with high bioenergetic demands such as heart, skeletal muscle, brown adipose, kidney, and brain suggests this receptor plays an important role in energy production. ERRα expression levels in those tissues have been shown to fluctuate with changing energy demands in response to physiological cues [9,10]. To fully understand the roles of ERRα in relation to human health, great effort has been devoted to searching for modulators of ERRα, including both synthetic compounds and those of natural origin [11,12,13,14,15,16,17].

Recently, overexpression of ERRα was found in cancer cells in various tissues, such as breast and colorectal tumors; unfortunately, this phenomenon is associated with a poor prognosis for these cancer types [18,19,20]. Furthermore, it has been shown that cancer cells can reprogram specific metabolic pathways to favor their unique bioenergetic requirements [21,22]. When ERRα is paired with a co-activator, peroxisome proliferator-activated receptor γ coactivator 1α (PGC-1α), a master regulator in energy homeostasis, its activity is greatly enhanced [23,24]. The PGC/ERR axis is not only regulating transcriptional activity of the metabolic gene network [25,26], but also influencing oncogenic signals that induce cell growth, proliferation, invasion, angiogenesis, and vascularization as demonstrated in a series of studies on the expression of vascular endothelial growth factor (VEGF) [27,28,29], hypoxia-inducible factor (HIF) [27,30,31], interleukin-6 (Il6) [32], and WNT11 [33,34]. Therefore, modulation of ERRα function may present a valuable therapeutic target to consider in developing novel drugs to treat cancer or diseases associated with metabolic disruptions.

In the Tox21 Program, an interagency collaboration among the National Institutes of Health (National Center for Advancing Translational Sciences; National Toxicology Program at the National Institute of Environmental Health Sciences), the Environmental Protection Agency, and the Food and Drug Administration, quantitative high-throughput screening (qHTS) approaches were developed [35,36,37] to screen a diverse 10,000 compound (10K) library that contains ~8900 unique compounds including environmental chemicals, natural dietary supplement products, pesticides, industrial compounds, and drugs, both active and withdrawn, for activation of many nuclear receptor (NR) signaling pathways, stress response pathways, and other targets. As part of this program, we previously developed, optimized, and validated assays to measure modulation of ERRα and PGC/ERRα signaling [13,14]; these assays were validated by screening the LOPAC library and then screened against the full Tox21 10K library, allowing for the identification of ERRα signaling activators. With these approaches, we identified ERRα and PGC/ERRα agonists based on compound structural similarity analysis [17]. In the present study, we have used these same approaches to identify compounds belonging to two distinct categories (drugs and pesticides) that function as antagonists to ERRα and the PGC/ERRα receptor pathway.

## 2. Results

### 2.1. qHTS Performance and Reproducibility

A primary qHTS of the Tox21 10K compound library was performed, using HEK293T cells transfected with an ERR vector, to identify environmental chemicals and drugs as potential ERR antagonists. The assay performance statistics, including a signal to background (S/B) ratio of 3.09 ± 0.16, a coefficient of variance (CV) of 2.96 ± 0.88%, and a Z’ factor of 0.71 ± 0.16, indicate a high level of technical performance for this ERR antagonist screen. Large screens with an S/B > 2, CV < 10%, and a Z’ factor between 0.5 and 1 are considered to be of good quality [38].

An evaluation of the reproducibility of the three independent 10K screening experiments was performed, as well as the Tox21-88 compound array, which was plated in duplicate on every library plate. For each of the three screens, results were used to bin compounds into one of three categories: active, inactive, or inconclusive; 18.43% of the compounds were classified as active (i.e., ERR antagonists) in the primary screen. After binning, the reproducibility was calculated based on the similarity in response (match rate) among the 3 runs for every compound. The ERR 10K antagonist triplicate run produced a mismatch rate of only 0.31%, indicating a robust performance for this assay. The Tox21-88 duplicate compounds also produced a low mismatch rate of 2.37%. Compounds identified as antagonists in the analysis of the primary 10K screening data were tested in a follow-up assay to confirm the initial results (Table 1 and Table 2). 

### 2.2. ERR Antagonists—Antineoplastic Agents

**Figure 1:**
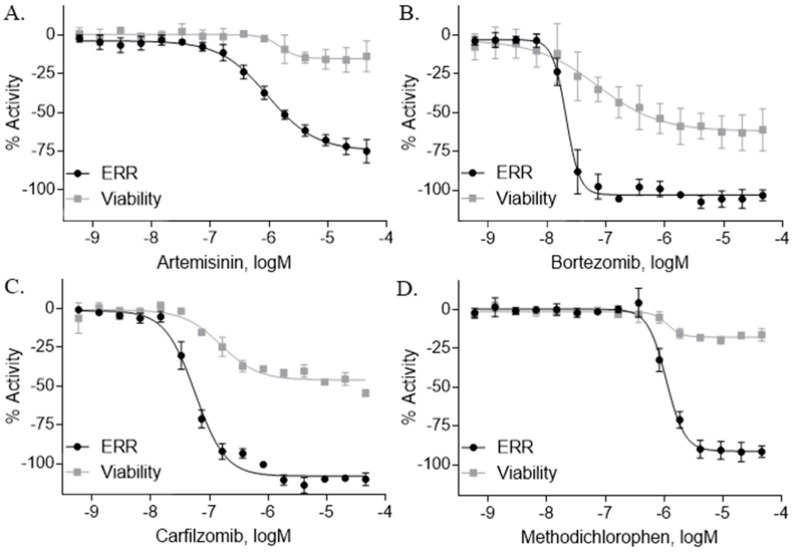
Concentration response curves of novel ERRα anticancer antagonists. Fifteen point dilutions, from the 10K ERRα antagonist and viability screen, of artemisinin (**A**), bortezomib (**B**), carfilzomib (**C**), and methodichlorophen (**D**) were completed. The ERR reporter cell line was treated with each compound for 18 h. The luminescence intensity was calculated after the addition of ONE-Glo to each well. The efficacy of each compound was then compared to the positive control, XCT790. Data are expressed as mean ± SD from triplicate experiments for each assay.

Seventy-eight compounds were identified, from the 10K screen, as ERRα antagonists by having an efficacy greater than −50% of the positive control, XCT790 (Supplementary Figure S1). Forty-five of the 78 compounds also had a potency value less than 10 µM; these were selected for additional investigation, based on their robust response. Within these 45, we recognized two major categories of compounds: antineoplastic agents and pesticides (Supplementary Figure S2). Nine antineoplastic agents exhibited ERR antagonism with an efficacy >−50% and a potency ranging from 17 nM to 10 µM in both the primary and the follow-up screens (Table 1). In the primary screen, the most potent compound was bortezomib (potency = 17 nM), a tubulin disrupter; efficacy of bortezomib was also high, at −92% (Table 1). The most efficacious antineoplastic compound in the primary screen was topotecan (a topoisomerase inhibitor), with a value of −127%; topotecan had a potency of 1.24 µM (Table 1). A viability assay was conducted concurrently with the ERR antagonist assay. Bortezomib had a cytotoxic effect in this assay with an IC_50_ of ~66 nM and an efficacy of −60.7%. However, the potency value for bortezomib in the viability assay is more than 3 times higher than the potency for bortezomib in the ERR antagonist assay, indicating that significant toxicity is seen only at a higher dose than is required for ERR antagonist activity (Table 1). Thus, the ERR antagonism (measured as loss of signal) seen with bortezomib is not the result of cytotoxicity (Figure 1B). Carfilzomib, a compound structurally similar to bortezomib, also demonstrated strong ERR antagonist activity, with an efficacy of −107% and a potency of 55 nM (Figure 1C). In the viability assay, carfilzomib showed an efficacy of <−50% and a potency >3-fold lower than the potency in the ERR antagonist assay (Table 1). Two other antineoplastic agents, artemisinin (Figure 1A) and methodichlorophen (Figure 1D), were identified as ERR antagonists with efficacies of −71.6% and −90.9% and potencies of 1.01 µM and 1.06 µM, respectively (Table 1). Neither compound demonstrated significant cytotoxicity, indicating that their ERR antagonist activities were not due to cytotoxicity (Table 1).

Due to the cooperative relationship between ERRα and PGC-1α, the ERR/PGC antagonist assay was also performed to compare each compound’s ERR antagonist activity in the presence and absence of the cofactor, PGC-1α. Artemisinin, bortezomib, carfilzomib, and methodichlorophen all exhibited antagonism in the ERR/PGC assay, implying these compounds inhibit ERR via PGC-1α (Table 1). Interestingly, SAHA and etoposide demonstrated antagonistic activity in the ERR assay while exhibiting agonist activity in the ERR/PGC assay (Supplementary Figures S3 and S4).

### 2.3. ERR Antagonists—Pesticides

**Figure 2:**
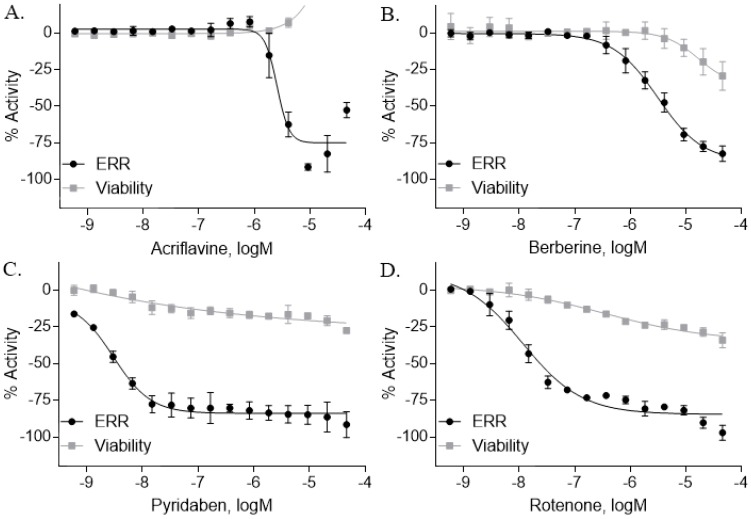
Concentration response curves of potential novel ERRα pesticide antagonists. Fifteen point titrations, from the 10K ERRα antagonist and viability screen, of acriflavine (**A**), berberine (**B**), pyridaben (**C**), and rotenone (**D**) were performed. The ERR reporter HEK293 cell line was treated with each compound for 18 h. The luminescence intensity was calculated after the addition of ONE-Glo to each well. The efficacy of each compound was then compared to the positive control, XCT790. Data were expressed as mean ± SD from triplicate experiments for each assay.

Another group of compounds categorized as ERR antagonists consisted of 13 pesticides (Table 2). Each of these compounds had an efficacy greater than −50% and a potency ranging from 4.5 nM to 6 µM. The most potent pesticide was fenpyroximate, which had a potency of 4.5 nM and was inactive in the viability assay, meaning there was an absence of any evidence of cytotoxicity (Table 2). Fenpyroximate also demonstrated ERR/PGC antagonist activity with an efficacy of −36.8% and potency of 8.8 nM in the primary screen (Table 2). Proflavin, inactive in the viability assay, was the most efficacious pesticide overall (−95% in the ERR assay and −94.9% in the ERR/PGC assay) (Table 2). The dose response curves for four representative pesticides (acriflavine, berberine, pyridaben, and rotenone) that were potent antagonists in the ERR assay are shown in Figure 2, along with their viability assay curves. Of these four, only berberine showed evidence of cytotoxicity (viability efficacy, −32%) (Figure 2). All four compounds also demonstrated antagonism in the ERR/PGC assay (Table 2).

### 2.4. Selectivity of ERR Antagonists

**Figure 3:**
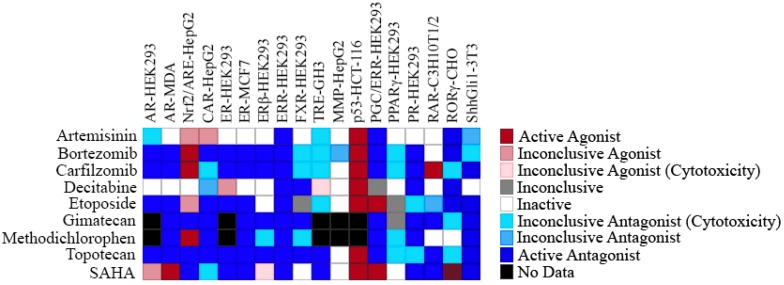
Assay heatmap of anticancer ERRα antagonists. The nine potential ERRα antagonist antineoplastic agents were screened, and their activity outcome in 15 antagonist assays (AR-HEK293, AR-MDA, CAR-HepG2, ER-HEK293, ER-MCF7, ERβ-HEK293, ERR-HEK293, FXR-HEK293, TRE-GH3, PGC/ERR-HEK293, PPARγ-HEK293, PR-HEK293, RAR-C3H10T1/2, RORγ-CHO, ShhGli1-3T3), 2 agonist assays (Nrf2/ARE-HepG2 and p53-HCT-116), and the MMP-HepG2 assay is displayed.

Due to the complicated signaling network and cross-talk that are involved in endocrine disruption, we examined the effect of the 22 antineoplastic agents and pesticides which were identified as ERR and ERR/PGC antagonists in 16 additional NR and signaling pathways. The activities of each of the antineoplastic agents are shown in Figure 3. All of these antineoplastic agents were active in the p53 assay, a finding that is consistent with the fact that many antineoplastic agents activate p53 [39] (Figure 3). Each antineoplastic compound, with the exception of decitabine, has antagonist activity in the sonic hedgehog assay (Figure 3); this signaling pathway was previously known to be associated with tumor development [40]. Artemisinin, bortezomib, carfilzomib, etoposide, and methodichlorophen were also active agonists in the Nrf2-ARE assay, which is a signaling pathway activated in response to reactive oxygen species (Figure 3). With the exception of artemisinin, each of the compounds also identified as a constitutive androstane receptor (CAR) signaling pathway antagonist (Figure 3).

**Figure 4:**
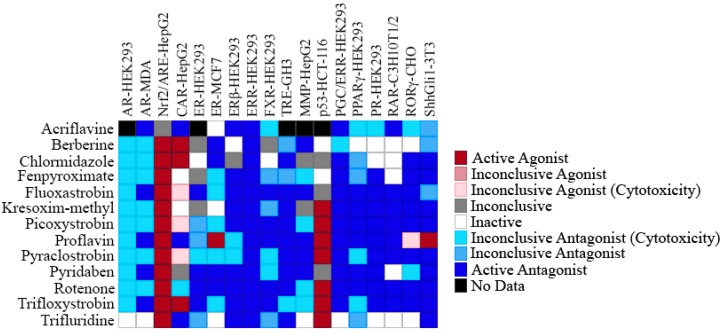
Assay heatmap of pesticide ERRα antagonists. The thirteen potential ERRα antagonist pesticide compounds were screened, and their activity outcome in 15 antagonist assays (AR-HEK293, AR-MDA, CAR-HepG2, ER-HEK293, ER-MCF7, ERβ-HEK293, ERR-HEK293, FXR-HEK293, TRE-GH3, PGC/ERR-HEK293, PPARγ-HEK293, PR-HEK293, RAR-C3H10T1/2, RORγ-CHO, ShhGli1-3T3), 2 agonist assays (Nrf2/ARE-HepG2 and p53-HCT-116), and the MMP-HepG2 assay is displayed.

The pesticide screening results for these multiple pathways are shown in Figure 4. Every compound except acriflavine (which was inconclusive) was active in the Nrf2-ARE assay, and seven of the pesticides also activated p53 (Figure 4). The overall activity pattern for this group of pesticides appears to involve nuclear receptor antagonism. Antagonist activity was seen in several NR assays, including the androgen receptor, estrogen receptor α, estrogen receptor β, farnesoid x receptor, thyroid hormone receptor β, peroxisome proliferator-activated receptor γ, progesterone receptor, retinoic acid receptor, and retinoic acid-related orphan receptor assays (Figure 4). These thirteen pesticides also appear to antagonize other signaling pathways, e.g., the sonic hedgehog and mitochondrial membrane potential pathways (Figure 4).

### 2.5. Effects of the ERR Antineoplastic Agents on Gene Expression

The antineoplastic agents tested in the current study were identified as ERR antagonists using a luciferase reporter assay. We conducted additional experiments to confirm this observed response by evaluating the effects that these compounds might have on the expression of ERR downstream genes including COX8α, IDH3α, PPARα, COX4I1 and cytochrome c. Five of the nine antineoplastic agents showed significant suppression of mRNA expression for at least one of the five downstream genes (Table 3). Artemisinin inhibited two of these endocrine disrupting genes (IDH3α: 30.2% and cytochrome c: 36.2%) (Table 3), while also suppressing the mRNA expression of ERRα itself (data not shown); no other antineoplastic agent showed inhibition of ERRα gene expression. Bortezomib inhibited expression of COX8α, IDH3α, and cytochrome c by 37.7%, 17.6%, and 48.3%, respectively. Carfilzomib, structurally similar to bortezomib, inhibited COX8α expression by 39.6% and cytochrome c by 44.2%, but not IDH3α (Table 3). Methodichlorophen also inhibited expression of multiple genes: expression of IDH3α, COX4I1, and cytochrome c was inhibited by 50.8%, 12.5%, and 47.6%, respectively (Table 3). Gimatecan inhibited expression only of cytochrome c (Table 3).

### 2.6. Effects of the Pesticide Antagonists on Gene Expression

Nine of the 13 pesticides significantly suppressed expression of at least one of the five downstream genes (Table 4). Acriflavine inhibited expression of IDH3α by 74.2% and cytochrome c by 41.8% (*p* < 0.001) (Table 4). Berberine suppressed expression of these same two genes (Table 4). Pyridaben decreased expression of IDH3α by 14.6% (Table 4). Rotenone inhibited expression of IDH3α by 22.4% (*p* < 0.01) and cytochrome c by 27.3% (*p* < 0.05) (Table 4).

## 3. Discussion

ERRα plays an important role in endocrine and energy homeostasis, and through these effects, may also have an important role in carcinogenesis [9,10,18,19,20]. Therefore, identifying ERRα antagonists may guide the development of novel therapeutic drugs, as well as uncover potential toxicities associated with drugs currently on the market. The current study identified five antineoplastic agents (artemisinin, bortezomib, carfilzomib, gimatecan, and methodichlorophen) and nine pesticides (acriflavine, berberine, chlormidazole, fluoxastrobin, picoxystrobin, proflavin, pyridaben, rotenone, and trifloxystrobin) that suppress ERRα activity in reporter gene and mRNA expression assays. Because the modes of action for these compounds are diverse and complex, it will require additional research to better understand the full range of their biological activity. This study represents the first step in characterizing their modes of action and their potential biological impact.

One group of compounds identified in our study as ERRα antagonists are antineoplastic agents. These compounds have also been identified as antagonists in several other nuclear receptor high-throughput screens conducted in our laboratory (Figure 3), findings that underscore the potentially broad reaching and complex network of pathways stimulated by these agents. However, it was determined that many of the ERRα antagonist antineoplastic agents were activators in the p53 assay. It had been previously shown that XCT790, a known ERRα inhibitor, can stimulate p53 expression [41]. It has also been demonstrated that ERRα plays a significant role in blocking methotrexate-induced reactive oxygen species production and is also involved in methotrexate resistance through the p53 apoptosis pathway [42]. Therefore, it is clear that a connection exists between ERRα and p53, and future work may further define their relationship. ERRα itself has a complex network of downstream target genes. Five of these genes (COX8a, IDH3, PPARa, COX411, and cytochrome c) were evaluated for inhibition of gene expression by these antineoplastic agents. The five antineoplastic agents listed above significantly inhibited expression of at least one of the five downstream genes that were studied, confirming a role for each of the compounds in suppressing ERRα signaling (Table 3). 

Artemisinin is a known anti-malarial drug [43,44] that also exhibits anticancer activity [45]. It induces apoptosis, changes in the expression of genes involved in cancer cell progression, and acts as an inhibitor to some histone deacetylase enzymes [46]. Our results (Figure 1A) show that artemisimin strongly inhibits ERRα reporter activity in both the ERR and PGC/ERR cell lines (Table 1), and significantly suppresses IDH3α, and cytochrome c mRNA expression (Table 3). One distinction of artemisimin’s action among the antagonist drugs tested in this study is its ability to inhibit ERRα expression at the transcriptional level (data not shown; [46]).

The first proteasome inhibitor to be developed, bortezomib (PS-341), which induces cell cycle arrest and apoptosis through tubulin disruption, was developed as an anticancer drug for Non-Hodgkin’s lymphoma [47]. Structurally similar compounds, such as carfilzomib, were developed for the treatment of multiple myeloma and myeloma-induced bone disease [48]. Intracellular levels of PGC-1α and ERRα are regulated by the proteasome system, which is particularly critical in effecting the rapid degradation of PGCs. It is predicted that these proteasome inhibitors will also affect ERRα and PGC-1α stability and thus their function. Despite this expectation, whether bortezomib and/or carfilzomib directly target ERRα or PGC-1α specifically, requires further investigation.

Methodichlorophen was promoted initially as an antitumor drug due to its action in inhibiting tetrahydrofolate reductase [49], but it is now used as a clinical diagnostic and treatment for corticosteroid hormone irregularities [50,51]. Gimatecan, a member of the camptothecin class of compounds, is a recently developed topoisomerase I inhibitor with anti-tumor properties [52,53]. It has been shown to be a potent alternative treatment in patients with resistant disease, including recurrent epithelial ovarian, fallopian tube, and peritoneal cancers, and has a manageable safety profile [54]. Based on this activity profile, it is reasonable to assume expanded applications in the future, increasing the need to identify any other therapeutic or toxicity pathways this compound might modulate. In the current study, we identified gimatecan as an ERRα antagonist through the suppression of cytochrome c (Table 3); this may represent a new therapeutic pathway for this drug. Given the diverse and numerous mechanisms of action of antineoplastic agents, any new biological activity information may prompt new approaches in treatment options.

Suberoylanilide hydroxamic acid (SAHA; vorinostat), a well-established anticancer drug [55], acts as a histone deacetylase inhibitor (HDAC) for HDAC classes I and II. SAHA is an NAD-independent and zinc-dependent enzyme [56,57,58]. It enhances the acetylation of all four core histones as well as many transcription factors [55]. These different HDACs form large multiprotein complexes with coactivators and corepressors, thereby variously promoting and inhibiting gene expression [59]. SAHA has also been shown to affect certain cellular functions critical to tumor growth, such as increasing expression of the cell cycle inhibitor p21^WAF1^ and inducing cell cycle arrest [60]. Since ERRα promotes cell growth and since increased expression of ERRα is an adverse marker for cancer progression, down regulation of ERRα activity/expression is sufficient to reduce the cancer cell population [30]; thus, it is reasonable to deduce that SAHA downregulates ERRα activity in the ERR cell line. In addition, the ERRα-specific inhibitor XCT790 (the reference compound in our antagonist assay), has recently been found to induce p21^WAF1^ expression while inhibiting cell growth [61], which further supports our reporter gene assay results showing that SAHA is an active antagonist in the ERRα cell line. In contrast, SAHA acts as a potent agonist in the PGC/ERRα cell line (Supplementary Figure S4). Recently, reversible acetylation of PGC-1α has emerged as a key mechanism for regulating the activity of this coactivator [62]. This mechanism acts as a sensor and effector to guarantee metabolic flexibility in normal cells; however, in some types of cancer cells only low levels of PGC-1α have been found (i.e., renal carcinoma) [63,64]. In a deactylated state, PGC-1α is activated by Sirt1, an NAD-dependent and zinc-independent enzyme (HDAC III class) [65]; however, SAHA, because it only affects HDACs containing zinc in their catalytic sites, has no effect on the activity of Sirt1 which has NAD in the catalytic site [65]. The mechanism through which SAHA stimulates PGC/ERR reporter activity is as yet unclear. One possible explanation is that SAHA has no effect on PGC-1α’s acetylated status but increases the acetylation of many other transcription factors, thereby modifying the chromatin structure in favor of an increase in gene expression. The multiprotein complexes involved in ERRα reporter activity in the PGC/ERR cell line will differ from the ERR cell line; instead of recruiting corepressors to the repressed chromatin structure and the transcriptional complex, SAHA recruits coactivators to an open chromatin structure in the PGC/ERR cell line and induces gene activation. Nonetheless, further study is required to clarify the mechanism of action for SAHA in the PGC/ERR cell line.

Etoposide, another of the antineoplastic agents identified as a modulator of ERRα and PGC/ERRα activity, forms a ternary complex with DNA and topoisomerase II, preventing religation of the broken DNA strand that is created during the process of DNA synthesis, thereby resulting in DNA strand breaks and cytotoxicity. This inhibition of topoisomerase II is the basis of its use as a chemotherapeutic agent [66]. Recent studies have shown that etoposide also has a high affinity for chromatin and histones, suggesting that it may affect chromatin organization and gene expression [66,67]. Etoposide was also reported to stimulate PGC-1α expression via AMPK activation and increase mitochondrial biogenesis [68]. Decreases in the number of mitochondria have been linked to neoplastic transformation [69]; thus, mitochondrial biogenesis via the PGC-1α pathway may play a critical role in tumor suppression [70]. Our screening data demonstrating increased reporter activity in the PGC/ERR cell line after etoposide treatment (Supplement Figure S1) is in agreement with etoposide’s ability to stimulate PGC-1α expression.

A second group of compounds identified as ERRα antagonists in the current study are pesticides. These compounds, similar to what was observed with the antineoplastic agents, also antagonize many of the same nuclear receptors (Figure 4). Interestingly, with the exception of acriflavine, all of the pesticides studied here activate the Nrf2/ARE signaling pathway (Figure 4). While activation of the Nrf2/ARE pathway counters oxidative stress and thus is beneficial to the cell [71], it is likely that these pesticides are inducing the formation of free radicals, which then promotes the translocation of Nrf2 into the nucleus and subsequent binding to the ARE [72,73]. In a study done by Zhou et al., it was shown that ERRβ but not ERRα inhibited transcriptional expression of Nrf2 [74]; thus far, to our knowledge, there is no study to indicate that Nrf2 and ERRα have a direct link. In addition to the NR reporter gene assays, gene expression studies were also conducted with the pesticides, and nine pesticides were shown to significantly inhibit the expression of at least one of the five ERRα downstream genes that we studied, providing additional evidence of their ERRα antagonist activities (Table 4). The pesticides identified in our assays as antagonists of ERRα and PGC-1α, acriflavine, berberine, chlormidazole, fluoxastrobin, picoxystrobin, proflavin, pyridaben, rotenone, and trifloxystrobin, inhibit and/or control pests by adversely affecting metabolism and mitochondrial function through a variety of different mechanisms [75,76,77,78,79,80,81,82,83,84]. The data derived from our study showed that the activity of ERRα and PGC-1α was down-regulated after exposure to these pesticides (Table 1, Table 2, Table 3 and Table 4). Since mitochondrial function is a major target of the ERR/PGC signaling pathways [85,86], this new information provides additional insight into the mechanism of action for these pesticides.

In conclusion, the Tox21 10K compound collection was screened for the identification of ERRα antagonists using the ERR and ERR/PGC reporter cell lines. We identified two groups of compounds, antineoplastic agents and pesticides, as ERRα antagonists in this study (Table 1 and Table 2). Five antineoplastic agents (artemisinin, bortezomib, carfilzomib, gimatecan, and methodichlorophen) and nine pesticides (acriflavine, berberine, chlormidazole, fluoxastrobin, picoxystrobin, proflavin, pyridaben, rotenone, and trifloxystrobin) were further confirmed as ERRα antagonists through gene expression studies that showed significant suppression of downstream ERRα target genes (Table 3 and Table 4). Due to the importance of the ERR signaling pathway in maintaining metabolic homeostasis as well as the role that ERRα plays in carcinogenesis, compounds that modulate ERRα activity deserve a thorough investigation of their biological activity. This study provides important insights into the broad range of biological activity that may be anticipated for some of these ERRα modulating compounds. Additional work is recommended to better understand the effects of the antineoplastic compounds that we identified as ERRα modulators and to explore the possibility that these compounds may have as yet undefined applications in the treatment of human diseases.

## 4. Materials and Methods

### 4.1. Tox21 Chemical Library

The Tox21 chemical library was comprised of about 10,500 compounds (7872 unique) gathered from commercial sources by the National Toxicology Program (NTP), the National Center for Advancing Translational Sciences (NCATS), and the Environmental Protection Agency (EPA). These chemical substances include pesticides, drugs, industrial, and food compounds and were selected for multiple criteria, including compounds with properties conducive to HTS (molecular weight, volatility, solubility, logP), possible and definite environmental hazards or exposure concerns, commercial availability, and cost. There were also 88 diverse compounds selected as internal controls to assess reproducibility and determine positional plate effects as previously reported [36].

### 4.2. Cell Lines and Culture Conditions

ERR and PGC/ERR reporter HEK293 cells were developed previously [14]. The ERR reporter HEK293 cells were cultured in high glucose DMEM medium (ThermoFisher Scientific, Inc., Waltham, MA, USA) supplemented with 10% fetal bovine serum (FBS; ThermoFisher Scientific, Inc.), 4 mM of l-glutamine (ThermoFisher Scientific, Inc.), 1 mM of sodium pyruvate (ThermoFisher Scientific, Inc.), 100 U/mL of penicillin and 100 mg/mL of streptomycin (ThermoFisher Scientific, Inc.). PGC/ERR reporter HEK293 cells were cultured in the same medium as the ERR reporter HEK293 cells with an additional 1 μg/mL puromycin (ThermoFisher Scientific, Inc.) as the selection marker for PGC-1α expression. 

HepG2 cells were purchased from American Type Culture Collection (ATCC, Manassas, VA, USA). HepG2 cells were cultured in Eagle’s Minimum Essential Medium (EMEM, ATCC) supplemented with 10% Hyclone™ FBS (ThermoFisher Scientific, Inc.), and 100 U/mL penicillin-100 µg/mL streptomycin. 

AR-HEK293, ARE-HepG2, ERα-HEK293, ERβ-HEK293, FXR-HEK293, p53-HCT-116, PPARγ-HEK293, and PR-HEK293 cells were purchased from ThermoFisher Scientific, Inc. These cell lines contain a β-lactamase reporter gene under control of the response elements for androgen receptor (AR), Nrf2/antioxidant response element (ARE), ERα, ERβ, farnesoid X receptor (FXR), p53, peroxisome proliferator-activated receptor gamma (PPARγ), and progesterone receptor (PR) that have been stably integrated into HEK293 (AR, ERα, ERβ, FXR, and PR), HEK293H (PPARγ), HepG2 (ARE), or HCT-116 (p53) cells. 

The CAR-HepG2 cell line was developed previously [37]. The CAR reporter cells were cultured in DMEM (ThermoFisher Scientific, Inc.) supplemented with 10% Hyclone™ FBS, (5 µg/mL blasticidin (ThermoFisher Scientific, Inc.), 0.5 mg/mL geneticin (ThermoFisher Scientific, Inc.), and 100 U/mL penicillin and 100 μg/mL streptomycin.

The MDA-kb2-AR cell line was purchased from the ATCC and was developed from the parental human breast cancer cell line MDA-MB-453. MDA-kb2-AR cells express firefly luciferase under control of the MMTV promoter that contains response elements for both glucocorticoid receptors (GR) and AR. MDA-kb2-AR cells were cultured in L-15 Medium (ATCC) supplemented with 10% FBS, and 100 U/mL penicillin-100 μg/mL streptomycin.

The ER-vMCF7 cell line was provided by Dr. Michael S. Denison (University of California, Davis). ER-vMCF7 cells endogenously express full-length ERα and are stably transfected with a plasmid containing four estrogen responsive elements (ERE) upstream of a luciferase reporter gene. These cells were cultured in MEMα medium (ThermoFisher Scientific, Inc.) supplemented with 10% Premium FBS (Atlanta Biologicals), 400 µg/mL G418, and 100 U/mL penicillin-100 μg/mL streptomycin.

TRE-GH3 cells, provided by Dr. Albertinka J. Murk (Wageningen University, The Netherlands) [87], stably express a modified firefly luciferase reporter gene under the regulation of a pair of thyroid hormone response elements (TRE). These cells were cultured in DMEM/F-12 (ThermoFisher Scientific, Inc.) supplemented with 10% FBS and 100 U/mL penicillin-100 µg/mL streptomycin.

RORγ-CHO cells were provided by Dr. Anton M. Jetten (NIEHS/NIH). These cells express a luciferase reporter gene under the control of a TET-inducible retinoid-related orphan receptor (ROR) expression factor and ROR response element. RORγ-CHO cells were cultured in F12 medium supplemented with 10% FBS approved for use with the Tet-on system (Clontech) and 100 U/mL penicillin-100 µg/mL streptomycin.

RAR-C3H10T1/2 [88] and ShhGli1-3T3 cell lines were provided by Drs. Yanling Chen and David H. Reese (FDA). The RAR-C3H10T1/2 cells contain a firefly luciferase gene under the control of the retinoic acid response element and were cultured in Eagle’s Basal Medium (Invitrogen) supplemented with 10% heat-inactivated FBS, 2 mM L-glutamine, 2 μg/mL puromycin and 100 U/mL penicillin-100 μg/mL streptomycin. The ShhGli1-3T3 cell line is an NIH/3T3–derived clone containing a firefly luciferase gene under the control of the Gli1 transcriptional response element. ShhGli1-3T3 cells were cultured in DMEM medium supplemented with 10% bovine calf serum (ATCC), 2 mM L-glutamine, 100 U/mL penicillin-100 μg/mL streptomycin, and 2 μg/mL puromycin.

All cells were cultured and maintained at 37 °C under a humidified atmosphere and 5% CO_2_, except the MDA-kb2-AR cells which were cultured and maintained at 37 °C under a humidified atmosphere and 0% CO_2_. All the cell culture reagents were obtained from ThermoFisher Scientific, Inc., except where mentioned above. All detailed descriptions of the assays are publicly available through the NCATS website (https://tripod.nih.gov/tox21/assays) and PubChem (https://pubchem.ncbi.nlm.nih.gov), while a detailed description of the cell lines is given in Supplementary Table S1.

### 4.3. ERR and PGC/ERR Reporter Assays

ERR or PGC/ERR reporter HEK293 cells suspended in culture medium without puromycin were dispensed at 2500 cells/5 μL/well in tissue culture–treated 1536-well white assay plates (Greiner Bio-One North America, Monroe, NC, USA) using a Thermo Scientific Multidrop Combi (ThermoFisher Scientific, Inc.). Each compound has been tested at 15 concentrations ranging from 1.2 nM to 92 μM in the primary screening. After the cells were incubated at 37 °C with 5% CO_2_ for 6 h, 23 nL of compounds or control, XTC790, was transferred into the assay plates using a Wako Pintool station (Wako Automation, San Diego, CA, USA). The assay plates were incubated at 37 °C for 18 h, followed by the addition of 5 μL ONE-Glo luciferase reagent (Promega, Madison, WI, USA) using a Flying Reagent Dispenser (Aurora Discovery, Carlsbad, CA, USA). After 30 min of incubation at room temperature, the luminescence intensity of the assay plates was quantified using a ViewLux plate reader (PerkinElmer, Shelton, CT, USA). The assays were performed three times for each compound concentration. These ERR and PGC/ERR reporter assays were multiplexed with the CellTiter-Fluor Cell Viability Assay (Promega), a fluorescence-based cell viability assay, to assess compound cytotoxicity.

### 4.4. AR-HEK293, ARE-HepG2, ER-HEK293, ERβ-HEK293, FXR-HEK293, p53-HCT116, PPARγ-HEK293, and PR-HEK293 β-Lactamase Reporter Gene Assays

AR-HEK293, ARE-HepG2, ERα-HEK293, ERβ-HEK293, FXR-HEK293, p53-HCT-116, PPARγ-UAS-293H, and PR-HEK293 cells were dispensed at 2,000 cells (AR, ARE, and ERβ), 3000 cells (PPARγ and PR), 4000 cells (P53), or 5000 cells (ERα and FXR) per well in 4 µL (ERβ and PR), 5 µL (Nrf2/ARE, FXR, PPARγ, and p53), or 6 µL (AR and ERα) of assay medium in 1536-well tissue culture treated black-well/clear bottom plates (Greiner Bio-One) using a Multidrop Combi dispenser. After the assay plates were incubated at 37 °C for 5–6 h, 23 nL of compounds dissolved in DMSO or positive controls were transferred to the assay plates via a Wako Pintool station (Wako). Except for ARE and p53, all other assays received 1 µL/well agonist (10 nM R1881 for AR; 0.5 and 5 nM β-estradiol for ERα and ERβ respectively; 300 µM CDCA for FXR; 50 nM Rosiglitazone for PPARγ; and 5 nM R5020 for PR) or assay medium using a Flying Reagent Dispenser (FRD). The assay plates were incubated for 16 h (AR, Nrf2/ARE, ERβ, FXR, p53, and PR), 17 h (PPARγ), or 18 h (ERα) at 37 °C, and 1 µL/well LiveBLAzer™ FRET-B/G CCF_4_-AM substrate (ThermoFisher Scientific, Inc.) detection mix was added using an FRD and the plates were incubated at room temperature for 2 h. The fluorescence intensity was measured by an Envision plate reader (PerkinElmer) at 405 nm excitation and 460 and 530 nm emissions. Data were expressed as the ratio of 460/530 nm emission values.

### 4.5. Mitochondrial Membrane Potential (MMP) Assay

HepG2 cells were dispensed at 2000 cells/well in 5 µL of culture medium in 1536-well tissue culture treated black wall/clear bottom plates (Greiner Bio-One) using a Multidrop Combi dispenser. After incubation at 37 °C for 18 h, 23 nL of the positive control or compounds dissolved in DMSO were transferred to the assay plates via a Wako Pintool station. The assay plates were incubated for 1 h at 37 °C, and 5 µL/well of Mito-MPS dye loading solution (Codex Biosolutions) was added using an FRD followed by another incubation for 30 min at 37 °C. The fluorescence intensity was measured by an EnVision plate reader (PerkinElmer) at 490 nm excitation and a 535 nm emission (for green monomers) as well as a 540 nm excitation with a 590 nm emission (for red aggregates). Data were expressed as the ratio of 590 nm/535 nm emission values.

### 4.6. AR-MDA, CAR-HepG2, ER-MCF7, RAR-C3H10T1/2, RORγ-CHO, ShhGli1-3T3, and TRE-GH3 Luciferase Reporter Gene Assays

AR-MDA, ER-vMCF7, RAR-C3H10T1/2, RORγ-CHO, ShhGli1-3T3, and TRE-GH3 cells were dispensed at 1000 cells (RAR-C3H10T1/2 and RORγ-CHO), 1500 cells (TRE-GH3), 2000 cells (ShhGli-3T3), 2500 cells (CAR-HepG2), 3000 cells (AR-MDA) or 4000 cells (ER-vMCF7) per well in 4 µL of the assay medium in 1536-well tissue culture treated white wall/solid bottom plates (Greiner Bio-One) using a Multidrop Combi dispenser. After the assay plates were incubated at 37 °C for 5–6 h (AR-MDA, CAR-HepG2, RORγ-CHO, ShhGli1-3T3, and TRE-GH3), 18 h (RAR-C3H10T1/2), or 24 h (ER-vMCF7), 23 nL of compounds dissolved in DMSO or positive controls were transferred to the assay plates via a Wako Pintool station. After the compound treatment, all of the assay plates for antagonist mode received 1 µL/well of agonist (0.5 nM R1881 for AR-MDA; 50 nM CITCO for CAR-HepG2; 0.1 nM β-Estradiol for ER-vMCF7; 1 µM retinol for RAR-C3H10T1/2; 1 µM doxycycline hyclate for RORγ-CHO; conditioned medium for ShhGli1-3T3; and 1 nM T3 for TRE-GH3) or assay medium using an FRD. The assay plates were then incubated for 6 h (RAR-C3H10T1/2); 16 h (AR-MDA and RORγ-CHO), 22 h (ER-vMCF7), or 24 h (CAR-HepG2, ShhGli1-3T3, and TRE-GH3) at 37 °C, and 4 µL/well of ONE-Glo™ Luciferase Assay reagent (Promega) was added using an FRD followed by an incubation at room temperature for 30 min. The luminescence intensity was measured by a ViewLux plate reader (Perkin Elmer). Data were expressed as relative luminescence units.

### 4.7. qHTS Data Analysis

The qHTS data were analyzed as described previously [36,89]. Each titration point was normalized relative to the positive control compound (XCT790 = −100%) and DMSO-only wells (0%) according to the following equation: % Activity = [(V_compound_ − V_DMSO_)/(V_DMSO_ − V_pos_)] × 100, where V_compound_ denotes the compound well values, V_pos_ denotes the median value of the positive control wells, and V_DMSO_ denotes the median values of the DMSO-only wells. The DMSO-only compound plates at the beginning and end of the compound plate stack were used to correct the data set by applying an in-house pattern correction algorithm [90]. The half maximum inhibition values (IC_50_) for each compound and maximum response (efficacy) values were obtained by fitting the concentration-response curves of each compound to a four-parameter Hill equation [91]. Compounds were designated as Class 1–4 according to the type of concentration–response curve observed [92,93]. For each reading, the activity outcome of a test compound was first categorized by the average curve rank from the triplicate runs and the reproducibility calls. The final activity outcome of each compound was based on the ERR antagonist readout activity and cell viability counter screen. Data reproducibility was categorized as an inactive match, active match, inconclusive, and mismatch according to the previously described criterion [36]. 

### 4.8. Quantitative Real-Time Polymerase Chain Reaction (qRT-PCR) 

Total RNA was isolated from treated ERR reporter cells using an RNeasy Mini Kit (Qiagen, Germantown, MD, USA), homogenized using a QIAshredder (Qiagen), and reverse transcribed with a High-Capacity RNA-to-cDNA™ Kit (ThermoFisher Scientific, Inc.) following the manufacturers’ instructions. Expression of ERRα, COX8α, IDH3α, PPARα, COX4I1, and cytochrome c genes was normalized against GAPDH. All primers were purchased from ThermoFisher Scientific, Inc. Real-time PCR assays were performed in 384-well plates on a QuantStudio 5 (Applied Biosystems, Foster City, CA, USA) with TaqMan™ Gene Expression Master Mix (ThermoFisher Scientific, Inc.). Induction values were calculated using the equation: Fold = 2^ΔΔ*C*t^, where ΔCt represents the differences in cycle threshold numbers between each of the target genes and GAPDH, and ΔΔCt represents the relative change in these differences between control and treatment groups. Experimental data are presented as a mean of triplicate determinations ± SD. Statistical comparisons were made by *t*-test, while the statistical significance was set at *p* values of <0.05 (light blue), <0.01 (medium blue), and <0.001 (dark blue).

## Figures and Tables

**Table 1:** Antineoplastic agents quantitative high-throughput screening (qHTS) primary and confirmation estrogen-related receptor (ERR) and peroxisome proliferator activated-receptor γ coactivator (PGC)/ERR half maximal effective concentration (EC_50_) and efficacy data.

Chemical Name (CAS #, Source) [Purity]	Structure	ERR-HEK293		PGC/ERR-HEK293	
		Primary EC_50_ (µM) [Efficacy (%)] Viability	Follow-Up EC_50_ (µM) [Efficacy (%)] Viability	Primary EC_50_ (µM) [Efficacy (%)] Viability	Follow-Up EC_50_ (µM) [Efficacy (%)] Viability
Artemisinin (63968-64-9, Microsource) [A]	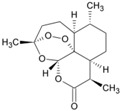	1.01 ± 0.297 [−71.6 ± 5.57] Inactive	2.44 ± 1.22 [−83.7 ± 14.6] Inactive	1.78 ± 0.401 [−57.6 ± 2.03] Inactive	14.5 ± 10.8 [−72.8 ± 22.0] Inactive
Bortezomib (179324-69-7, Selleck) [N/A]	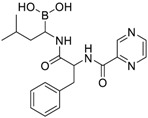	0.0170 ± 0.00781 [−92.2 ± 11.8] 0.0656 ± 0.0247 [−60.7 ± 7.59]	0.0167 ± 0.00608 [−125 ± 11.0] 0.0780 ± 0.0140 [−61.3 ± 6.93]	0.0169 ± 0.00554 [−92.2 ± 11.8] 0.0628 ± 0.0235 [−48.6 ± 1.34]	0.00962 ± 0.00452 [−116 ± 12.7] 0.0643 ± 0.0110 [−61.1 ± 3.07]
Carfilzomib (868540-17-4, Sequoia) [A]	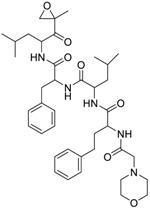	0.0552 ± 0.00374 [−107 ± 2.77] 0.178 ± 0.0767 [−46.9 ± 4.15]	0.0617 ± 0.00786 [−126 ± 10.9] 0.274 ± 0.0186 [−55.3 ± 0.231]	0.0615 ± 0.0198 [−121 ± 7.36] 1.36 ± 1.73 [−61.8 ± 18.0]	0.189 ± 0.0396 [−150 ± 8.02] 0.471 ± 0.0541 [−68.5 ± 2.09]
Decitabine (2353-33-5, Tocris) [A]	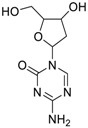	1.52 ± 0.348 [−79.1 ± 6.79] Inactive	3.10 ± 0.558 [−97.8 ± 15.4] Inactive	Inactive Inactive	Inactive Inactive
Etoposide (33419-42-0, Light Biologicals) [A]	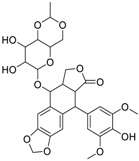	9.24 ± 8.56 [−115 ± 6.85] 56.3 ± 7.18 [−40.5 ± 13.0]	8.12 ± 4.42 [−88.9 ± 6.62] Inactive	2.10 ± 0.526 [62.9 ± 2.71] 56.3 ± 7.18 [−44.2 ± 8.70]	10.7 ± 2.44 [89.3 ± 19.5] Inactive
Gimatecan (292618-32-7, GVK) [I]	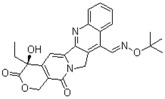	0.0497 ± 0.00894 [−117 ± 14.5] Inactive	0.0442 ± 0.0204 [−145 ± 16.0] Inactive	2.53 ± 1.92 [−115 ± 2.20] Inactive	3.34 ± 2.29 [−130 ± 20.6] Inactive
Methodichlorophen (7761-45-7, GVK) [A]	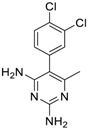	1.06 ± 0.122 [−90.9 ± 4.94] Inactive	1.60 ± 0.104 [−104 ± 7.68] Inactive	0.845 ± 0.0971 [−68.4 ± 7.64] Inactive	1.57 ± 0.354 [−61.5 ± 12.6] Inactive
Topotecan hydrochloride (119413-54-6, Prestwick) [A]	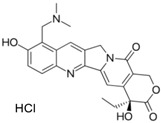	1.24 ± 0.0837 [−127 ± 11.8] Inactive	0.722 ± 0.285 [−140 ± 24.4] Inactive	14.1 ± 1.15 [−69.1 ± 5.82] Inactive	5.26 ± 0.00 [−78.8 ± 7.20] Inactive
Vorinostat (SAHA) (149647-78-9, Prestwick) [A]	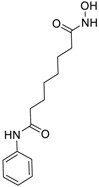	0.162 ± 0.0105 [−54.7 ± 3.05] 5.16 ± 0.879 [−33.1 ± 1.56]	0.359 ± 0.0234 [−55.0 ± 10.8] Inactive	2.11 ± 0.00 [382 ± 23.2] Inactive	2.44 ± 0.165 [479± 63.2] Inactive

Inactive = compounds with an efficacy less than 30 µM; ND = not determined; A = MW confirmed, purity > 90%; I = isomers; two or more isomers detected; data are expressed as mean ± SD from triplicate experiments for each assay.

**Table 2:** Pesticide compound qHTS primary and confirmation ERR and PGC/ERR EC_50_ and efficacy data.

Chemical Name (CAS #, Source) [Purity]	Structure	ERR-HEK293		PGC/ERR-HEK293	
		Primary EC_50_ (µM) [Efficacy (%)] Viability	Follow-Up EC_50_ (µM) [Efficacy (%)] Viability	Primary EC_50_ (µM) [Efficacy (%)] Viability	Follow-Up EC_50_ (µM) [Efficacy (%)] Viability
Acriflavine hydrochloride (69235-50-3, Microsource) [I]	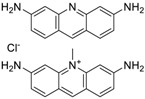	2.75 ± 0.885 [−83.8 ± 13.1] Inactive	8.96 ± 2.65 [−153 ± 1.29] Inactive	2.30 ± 0.316 [−50.7 ± 8.37] Inactive	5.66 ± 3.31 [−132 ± 25.7] Inactive
Berberine (633-65-8, Labotest) [A]	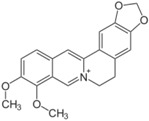	3.16 ± 0.713 [−88.4 ± 7.45] 12.8 ± 0.837 [−31.7 ± 10.2]	3.29 ± 2.27 [−92.0 ± 16.0] 12.7 ± 5.33 [−54.1 ± 10.6]	6.13 ± 1.71 [−77.3 ± 14.6] 17.3 ± 4.82 [−31.8 ± 3.57]	8.93 ± 4.01 [−87.4 ± 20.8] 17.3 ± 1.17 [−43.4 ± 9.71]
Chlormidazole (3689-76-7, Vitas) [A]	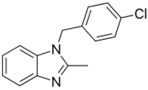	0.784 ± 0.0999 [−64.3 ± 5.95] Inactive	2.31 ± 2.55 [−96.2 ± 32.3] 19.3 ± 6.20 [−43.6 ± 15.3]	1.48 ± 1.02 [−42.2 ± 2.79] Inactive	0.565 ± 0.352 [−56.9 ± 16.8] Inactive
Fenpyroximate (111812-58-9, Sigma) [N/A]	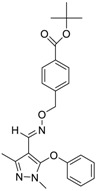	0.00452 ± 0.000577 [−67.1 ± 9.31] Inactive	0.00699 ± 0.00328 [−89.6 ± 6.08] 51.4 ± 87.2 [−36.9 ± 12.9]	0.00875 ± 0.000572 [−36.8 ± 2.17] Inactive	0.0186 ± 0.0210 [−56.2 ± 10.5] Inactive
Fluoxastrobin (361377-29-9, Sigma) [N/A]	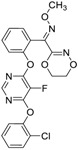	2.05 ± 0.893 [−90.0 ± 16.2] 22.5 ± 11.2 [−49.1 ± 7.27]	7.19 ± 3.10 [−109 ± 19.7] 24.1 ± 12.2 [−46.2 ± 5.55]	14.0 ± 12.0 [−77.2 ± 11.6] 38.5 ± 13.8 [−46.4 ± 6.61]	7.62 ± 2.72 [−90.2 ± 12.1] 41.3 ± 2.69 [−43.4 ± 6.25]
Kresoxim-methyl (143390-89-0, Light Biologicals) [A]	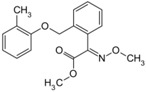	5.65 ± 6.96 [−86.6 ± 21.1] Inactive	8.31 ± 3.29 [−102 ± 1.40] Inactive	7.66 ± 6.87 [−56.7 ± 8.51] Inactive	17.7 ± 6.04 [−61.1 ± 10.0] Inactive
Picoxystrobin (117428-22-5, Sigma) [N/A]	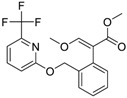	0.807 ± 0.279 [−83.9 ± 10.6] Inactive	3.42 ± 0.393 [−86.7 ± 15.8] 32.1 ± 14.8 [−30.1 ± 2.49]	2.79 ± 2.40 [−44.2 ± 4.37] Inactive	4.19 ± 2.97 [−43.6 ± 14.2] Inactive
Proflavin hemisulfate (1811-28-5, Vitas^1^) [A]	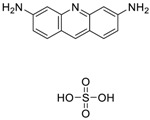	2.89 ± 0.398 [−95.0 ± 10.9] Inactive	3.38 ± 0.772 [−86.8 ± 8.82] Inactive	20.9 ± 16.2 [−94.9 ± 12.3] Inactive	1.88 ± 2.61 [−39.0 ± 79.4] Inactive
Pyraclostrobin (175013-18-0, Sigma) [A]	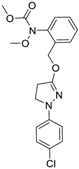	0.239 ± 0.130 [−90.7 ± 11.9] 40.9 ± 17.8 [−31.0 ± 2.62]	0.762 ± 0.00 [−80.0 ± 5.67] Inactive	0.838 ± 0.771 [−58.2 ± 4.17] 74.5 ± 10.3 [−31.3 ± 5.61]	0.539 ± 0.760 [−42.1 ± 9.82] Inactive
Pyridaben (96489-71-3, Sigma) [A]	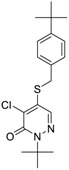	0.00592 ± 0.000815 [−77.3 ± 4.10] Inactive	0.0245 ± 0.00942 [−94.1 ± 13.4] Inactive	0.00781 ± 0.00301 [−57.6 ± 8.49] Inactive	0.0434 ± 0.0200 [−63.1 ± 29.7] Inactive
Rotenone (83-79-4, Microsource) [B]	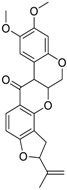	0.0133 ± 0.00476 [−92.4 ± 3.03] Inactive	0.0160 ± 0.00671 [−95.8 ± 17.8] 0.429 ± 0.168 [−50.4 ± 7.00]	0.00742 ± 0.00207 [−31.0 ± 4.14] 1.01 ± 0.454 [−35.8 ± 12.3]	0.0618 ± 0.0610 [−41.9 ± 8.70] 2.97 ± 0.341 [−52.4 ± 5.35]
Trifloxystrobin (141517-21-7, Sigma) [A]	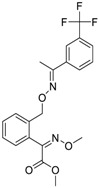	0.801 ± 0.0542 [−88.1 ± 18.7] Inactive	4.36 ± 0.998 [−100 ± 4.87] Inactive	2.30 ± 0.187 [−45.2 ± 1.61] Inactive	5.86 ± 0.807 [−56.7 ± 10.7] Inactive
Trifluridine (70-00-8, Prestwick) [A]	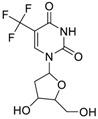	0.875 ± 0.0593 [−51.8 ± 5.77] Inactive	1.75 ± 0.314 [−67.7 ± 6.20] Inactive	Inactive Inactive	Inactive Inactive

Inactive = compounds with an efficacy less than 30 µM; A = MW confirmed, purity > 90%; B = MW confirmed, purity 75%–90%; I = isomers; two or more isomers detected; data are expressed as mean ± SD from triplicate experiments for each assay.

**Table 3:** Percent inhibition of mRNA gene expression of antineoplastic agents.

	COX8α	IDH3α	PPARα	COX4I1	Cytochrome C
XCT790 (12 µM)	65.0 ± 8.41	42.6 ± 6.59	16.0 ± 1.70	46.0 ± 2.16	60.8 ± 2.16
Artemisinin (^°^: 2 µM)(*: 7 µM)	39.0^°^ ± 15.1	30.2^*^ ± 4.28	26.2* ± 8.02	20.5* ± 4.06	36.2* ± 3.38
Bortezomib (20 µM)	37.7 ± 5.50	17.6 ± 4.66	14.0 ± 5.17	35.3 ± 3.49	48.3 ± 1.92
Carfilzomib (60 nM)	39.6 ± 1.81	1.73 ± 0.76	7.39 ± 4.61	37.8 ± 5.12	44.2 ± 0.70
Decitabine (2 µM)		5.79 ± 45.6			
Etoposide (18 µM)		7.19 ± 23.5			6.55 ± 3.18
Gimatecan (^°^: 0.2 µM)(*: 1 µM)	9.84^°^ ± 0.47			7.43* ± 4.35	31.5* ± 5.35
Methodichlorophen (2 µM)	17.1 ± 29.5	50.8 ± 8.69		12.5 ± 2.60	47.6 ± 7.45
Topotecan (^°^: 1.5 µM)(*: 7 µM)	23.6^*^ ± 13.6				18.1^°^ ± 9.85
SAHA (300 nM)	33.4 ± 22.5	26.1 ± 32.9			

mRNA levels were determined relative to the levels of GAPDH and normalized to the levels of vehicle control; (DMSO) and listed as % inhibition where DMSO = 0%; values are listed as mean ± S.D.; dark-blue highlight = *p* < 0.001, medium-blue highlight = *p* < 0.01, light-blue highlight = *p* < 0.05, no highlight = *p* > 0.05, blank = no inhibition.

**Table 4:** Percent inhibition of mRNA gene expression of pesticide compounds.

	COX8α	IDH3α	PPARα	COX4I1	Cytochrome C
XCT790 (12 µM)	65.0 ± 8.41	42.6 ± 6.59	16.0 ± 1.70	46.0 ± 2.16	60.8 ± 2.16
Acriflavine (3 µM)	30.2 ± 18.2	74.2 ± 9.92		4.76 ± 8.24	41.8 ± 3.72
Berberine (20 µM)	5.15 ± 0.17	15.1 ± 2.97		20.5 ± 4.06	46.2 ± 2.61
Chlormidazole (5 µM)		23.8 ± 3.17		4.71 ± 5.09	35.8 ± 3.92
Fenpyroximate (7.5 nM)					5.28 ± 4.56
Fluoxastrobin (7 µM)		13.3 ± 2.44			25.5 ± 7.81
Kresoxim-methyl (2 µM)	9.70 ± 12.8	8.26 ± 19.3			9.31 ± 0.48
Picoxystrobin (^°^: 1 µM)(*: 1.5 µM)	28.4^°^ ± 6.07	17.7^°^ ± 34.4		3.06* ± 0.28	31.6* ± 3.25
Proflavin (3 µM)	34.8 ± 26.7	24.9 ± 20.6			20.6 ± 8.69
Pyraclostrobin (1 µM)		5.06 ± 5.03			11.0 ± 8.54
Pyridaben (^°^: 5 nM)(*: 15 nM)	26.8^°^ ± 23.4	14.6* ± 8.15			20.3* ± 2.05
Rotenone (175 nM)		22.4 ± 3.14		2.34 ± 3.78	27.3 ± 7.33
Trifloxystrobin (0.5 µM)	38.4 ± 17.2	10.4 ± 0.21			
Trifluridine (1 µM)	43.2 ± 21.8				

mRNA levels were determined relative to the levels of GAPDH and normalized to the levels of vehicle control; (DMSO) and listed as % inhibition where DMSO = 0%; values are listed as mean ± S.D.; dark-blue highlight = *p* < 0.001, medium-blue highlight = *p* < 0.01, light-blue highlight = *p* < 0.05, no highlight = *p* < 0.05, blank = no inhibition.

## Supplementary Materials

The following are available online, Table S1: Cell Line Details; Figure S1. Chemical structure of XCT790; Figure S2. Flow chart of ERRα antagonist determination; Figure S3. Concentration response curves, using 15-point dilutions, were acquired on Etoposide using ERR, PGC/ERR, and viability assays; Figure S4. Concentration response curves, using 15-point dilutions, were acquired on SAHA using ERR, PGC/ERR, and viability assays.
