# Supplementary material for: Identification of Compounds That Inhibit Estrogen-Related Receptor Alpha Signaling Using High-Throughput Screening Assays
Source: Molecules. 2019 Feb 27;24(5):841. doi: 10.3390/molecules24050841 (PMC6429183; doi:10.3390/molecules24050841)
Supplement: Supplementary file 1 [file molecules-24-00841-s001.pdf]

Supplemental Material

Identification of compounds that inhibit estrogen-related receptor alpha signaling using high-throughput screening assays

Caitlin Lynch, Jinghua Zhao, Srilatha Sakamuru, Li Zhang, Ruili Huang, Kristine L. Witt, B. Alex Merrick, Christina T. Teng, Menghang Xia

Table of Contents

Tables

Table S1. Cell Line Details 2

Figures

Figure S1. Chemical structure of XCT790 3

Figure S2. Flow chart of ERRα antagonist determination 3

Figure S3. Concentration response curves, using 15-point dilutions, were acquired on Etoposide using ERR, PGC/ERR, and viability assays 4

Figure S4. Concentration response curves, using 15-point dilutions, were acquired on SAHA using ERR, PGC/ERR, and viability assays 4

Supplementary Table S1. Cell Line Details

Name	Cell Line	Target	Assay Readout	Acquired
AR-HEK293	HEK293	Androgen Receptor	Fluorescence	Life Technologies
AR-MDA	MDA-MB-453	Androgen Receptor	Luminescence	ATCC
Nrf2/ARE-HepG2	HepG2	Antioxidant Response Element	Fluorescence	Life Technologies
CAR-HepG2	HepG2	Constitutive Androstane Receptor	Luminescence	Dr. Caitlin Lynch<br>Dr. Hongbing Wang
ER-HEK293	HEK293	Estrogen Receptor α	Fluorescence	Life Technologies
ER-MCF7	vMCF7	Estrogen Receptor α	Luminescence	Dr. Michael S. Denison
ERβ-HEK293	HEK293	Estrogen Receptor β	Fluorescence	Life Technologies
ERR-HEK293	HEK293	Estrogen-Related Receptor α	Luminescence	Dr. Christina T. Teng
FXR-HEK293	HEK293	Farnesoid X Receptor	Fluorescence	Life Technologies
TRE-GH3	GH3	Thyroid Hormone Receptor	Luminescence	Dr. Albertinka J. Murk
MMP-HepG2	HepG2	Mitochondrial Membrane Potential	Membrane Potential	ATCC
p53-HCT-116	HCT-116	p53	Fluorescence	Life Technologies
PGC/ERR-HEK293	HEK293	Estrogen-Related Receptor α	Luminescence	Dr. Christina T. Teng
PPARγ-HEK293	HEK293H	Peroxisome Proliferator-Activated Receptor γ	Fluorescence	Life Technologies
PR-HEK293	HEK293	Progesterone Receptor	Fluorescence	Life Technologies
RAR-C3H10T1/2	C3H10T1/2	Retinoic Acid Receptor	Luminescence	Dr. Yanling Chen<br>Dr. David H. Reese
RORγ-CHO	CHO	Retinoic Acid-Related Orphan Receptor γ	Luminescence	Dr. Anton M. Jetten
ShhGli1-3T3	NIH3T3	Sonic Hedgehog Pathway	Luminescence	Dr. Yanling Chen<br>Dr. David H. Reese

Supplementary Figures

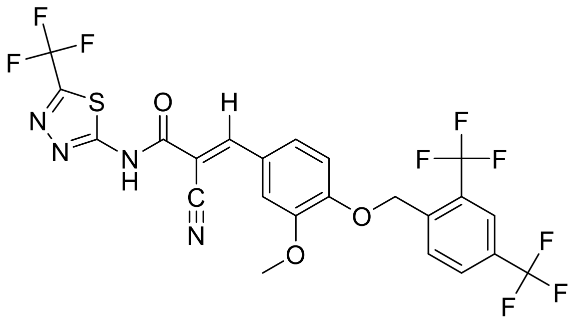

Figure S1. Chemical structure of XCT790.

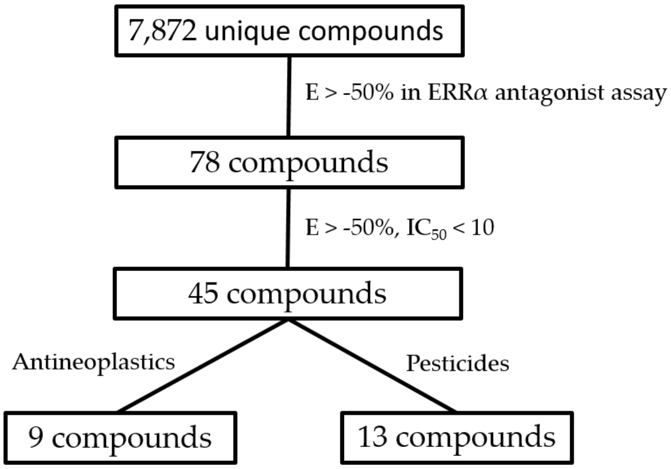

Figure S2. Flow chart of ERRα antagonist determination.

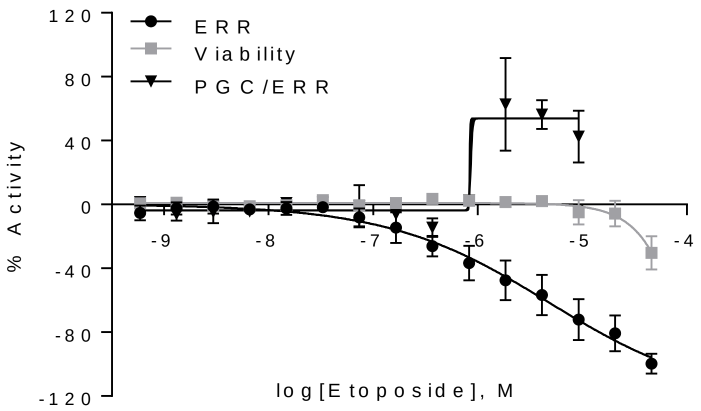

Figure S2. Concentration response curves, using 15-point dilutions, were acquired on Etoposide using ERR, PGC/ERR, and viability assays. The positive controls for each assay were made equal to 100%, and etoposide's data was compared to these numbers. Data were collected from the primary screenings and expressed as mean ± SD from triplicate experiments.

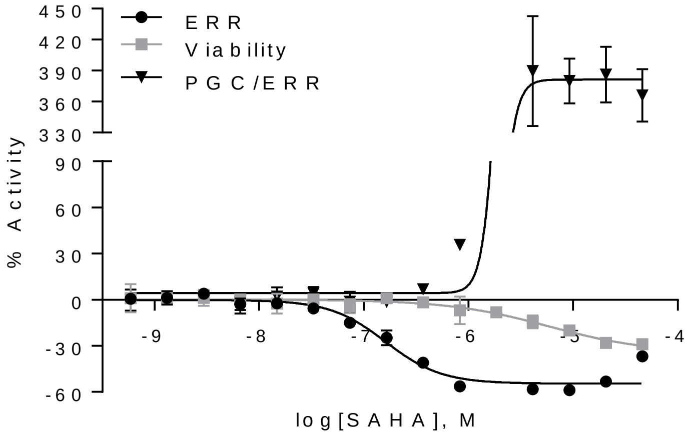

Figure S3. Concentration response curves, using 15-point dilutions, were acquired on SAHA using ERR, PGC/ERR, and viability assays. The positive controls for each assay were made equal to 100%, and SAHA's data was compared to these numbers. Data were collected from the primary screenings and expressed as mean ± SD from triplicate experiments.
